# The Elevated Pre-Treatment C-Reactive Protein Predicts Poor Prognosis in Patients with Locally Advanced Rectal Cancer Treated with Neo-Adjuvant Radiochemotherapy

**DOI:** 10.3390/diagnostics10100780

**Published:** 2020-10-02

**Authors:** Richard Partl, Katarzyna Lukasiak, Eva-Maria Thurner, Wilfried Renner, Heidi Stranzl-Lawatsch, Tanja Langsenlehner

**Affiliations:** 1 Department of Therapeutic Radiology and Oncology, Comprehensive Cancer Center, Medical University of Graz, 8036 Graz, Austria; katarzyna.lukasiak@klinikum-graz.at (K.L.); Ethurner86@gmail.com (E.-M.T.); heidi.stranzl@medunigraz.at (H.S.-L.); tanja.langsenlehner@medunigraz.at (T.L.); 2 Clinical Institute of Medical and Chemical Laboratory Diagnostics, Medical University of Graz, 8036 Graz, Austria; wilfried.renner@medunigraz.at

**Keywords:** locally advanced rectal cancer, neo-adjuvant radiochemotherapy, biomarker, inflammation, C-reactive protein (CRP), prognostic factor, outcome

## Abstract

The aim of the present study was to investigate the association of the pre-treatment C-reactive protein (CRP) plasma level with survival outcomes in a cohort of 423 consecutive patients with locally advanced rectal cancer treated with neo-adjuvant radiochemotherapy followed by surgical resection. To evaluate the prognostic value of the CRP level for clinical endpoints recurrence-free survival (RFS), local-regional control (LC), metastases-free survival (MFS), and overall survival (OS), uni- and multivariate Cox regression analyses were applied, and survival rates were calculated using Kaplan–Meier analysis. The median follow-up time was 73 months. In univariate analyses, the pre-treatment CRP level was a significant predictor of RFS (hazard ratio (HR) 1.015, 95% CI 1.006–1.023; *p* < 0.001), LC (HR 1.015, 95% CI 1.004–1.027; *p* = 0.009), MFS (HR 1.014, 95% CI 1.004–1.023; *p* = 0.004), and OS (HR 1.016, 95% CI 1.007–1.024; *p* < 0.001). Additionally, univariate analysis identified the MRI circumferential resection margin (mrCRM) and pre-treatment carcinoembryonic antigen (CEA) as significant predictor of RFS (HR 2.082, 95% CI 1.106–3.919; *p* = 0.023 and HR 1.005, 95% CI 1.002–1.008; *p* < 0.001). Univariate analysis also revealed a significant association of the mrCRM (HR 2.089, 95% CI 1.052–4.147; *p* = 0.035) and CEA (HR 1.006, 95% CI 1.003–1.008; *p* < 0.001) with MFS. Age and CEA were prognostic factors for OS (HR 1.039, 95% CI 1.013–1.066; *p* = 0.003 and HR 1.005, 95% CI 1.002–1.008; *p* < 0.001). In multivariate analysis that included parameters with a *p*-level < 0.20 in univariate analysis, the pre-treatment CRP remained a significant prognostic factor for RFS (HR 1.013, 95%CI 1.001–1.025; *p* = 0.036), LC (HR 1.014, 95% CI 1.001–1.027; *p* = 0.031), and MFS (HR 1.013, 95% CI 1.000–1.027; *p* = 0.046). The results support the hypothesis that an elevated pre-treatment CRP level is a predictor of poor outcome. If confirmed by additional studies, this easily measurable biomarker could contribute to the identification of patients who might be candidates for more aggressive local or systemic treatment approaches or the administration of anti-inflammatory drugs.

## 1. Introduction

Colorectal cancer (CRC) is one of the most commonly occurring cancers worldwide with a remarkable increase in incidence among adults younger than 50 years [1,2]. Approximately 35% of CRC diagnoses are located distal to the recto-sigmoid junction and specified as rectal cancer [3]. Combined-modality therapy in the setting of concurrent fluoropyrimidine-based chemotherapy with radiation to the pelvis followed by surgical resection has become the standard of care in locally advanced rectal cancer stages II and III [3,4]. Previous studies have compared preoperative versus postoperative radiochemotherapy (RCT) demonstrating that preoperative therapy is associated with a significant reduction in local recurrence and treatment-associated toxicity, although OS is similar in both groups. Moreover, some studies have indicated that preoperative radiotherapy (RT) or RCT is associated with increased rates of sphincter preservation [5,6,7]. For instance, Crane et al. demonstrated that complete response to neo-adjuvant radiochemotherapy (nRCT) enhanced the surgeon’s ability to achieve sphincter-preservation among patients with tumors within three centimeters of the dentate line [6]. In a previous analysis by our study group, we analyzed further patient- and tumor-associated routine parameters in low rectal cancer affecting the sphincter-preserving surgery rate. The parameters age, lymphocyte count and interval between nRCT and surgery at the beginning of an oncologic treatment contributed to the sphincter-preservation rate and could contribute to the identification of patients who could benefit from a more aggressive treatment approach [8].

The prognostication of the treatment response and the clinical outcome using clinical parameters may provide clinicians with additional information for a more individualized treatment approach. In the past, clinical parameters such as cT and cN stage have been found to be predictive of treatment response. More recently, various molecular biomarkers including gene expression profiles, DNA methylation, and miRNA patterns have been proposed as predictors of response to nRCT; however, determination of these parameters is associated with time-consuming procedures and laboratory efforts [1,2,3]. Therefore, the establishment of easily assessable and available pre-treatment prognostic biomarkers is warranted and intensively studied [9].

The biomarker C-reactive protein (CRP) is an acute-phase protein mainly produced in the liver in response to inflammation [10]. Plasma CRP has been proposed as a sensitive serological surrogate parameter for elevated levels of pro-inflammatory cytokines stimulating angiogenesis, tumor proliferation, and growth and represents an easily measurable, blood-based biomarker routinely analyzed before the initiation of treatment [11,12]. There is also evidence suggesting that CRP is not only a marker of inflammation but also plays an active role in regulating the tumor microenvironment and tumor cell growth and survival [13]. 

Chronic inflammation is a critical component of tumor development and progression. It may be present before a malignant change occurs predisposing individuals to cancer, conversely, an oncogenic change may induce an inflammatory microenvironment that promotes tumor development. Regardless of its origin, inflammation in the tumor microenvironment contributes to the proliferation and survival of malignant cells, promotes angiogenesis and metastasis, and alters responses to hormones and chemotherapeutic agents [12,14]. During chronic inflammation, the cytokines tumor necrosis factor (TNF-α), interleukin 6 (IL-6), transforming growth factor β (TGF-β), and interleukin 10 (IL-10) have been shown to be involved in tumor induction, proliferation, and metastasis [15]. CRP stimulates the production of inflammatory mediators including IL-1β, TNF-α, and reactive oxygen species. In addition, CRP induces E-selectin and vascular cell adhesion molecule (VCAM) expression, which are key players in the mediation of tumor cell adhesion to endothelial cells and transendothelial migration [16,17]. In tumors with a high local immune infiltration of FOXP3+ cells in the tumor microenvironment, more favorable survival outcomes could be observed [18,19]. However, little information is available about the relationship between local immune infiltration in the tumor microenvironment and the systemic inflammation. Gunnarsson et al. analyzed the relationship between CRP and the inflammatory microenvironment in colorectal cancer and identified an inverse correlation between CRP level and intratumor stroma infiltration by T-regulatory FOXP3+ immune cells [20].

Various previous studies demonstrate an association between the pre-treatment plasma CRP level and prognostic outcome in different cancer entities, including CRC [21,22,23]. However, data on the prognostic role of the pre-treatment CRP level in non-metastatic rectal cancer are limited and derived from studies analyzing relatively small numbers of patients. Toiyama et al. evaluated the prognostic role of the pre-treatment plasma CRP level in a cohort of 84 rectal cancer patients and demonstrated that an elevated CRP level predicted poorer OS and early recurrence in patients with locally advanced rectal cancer treated with nRCT followed by total mesorectal excision (TME) [24]. In contrast, Buijsen et al., who investigated various pre-treatment biomarkers as predictive factors for tumor response after nRCT in rectal cancer patients, did not detect a significant association between the pre-treatment CRP level and tumor response after nRCT [25].

The aim of the present study was to investigate the association of the pre-treatment CRP level with recurrence-free survival (RFS), loco-regional control (LC), metastases-free survival (MFS), and overall survival (OS) in order to validate and further clarify the prognostic significance of the CRP level in a large European cohort of locally advanced rectal cancer patients treated with nRCT followed by definitive surgery. 

## 2. Materials and Methods

A total of 540 consecutive cases with histologically confirmed locally advanced primary rectal cancer referred for nRCT from 2004–2015 at the Department of Therapeutic Radiology and Oncology were evaluated retrospectively. Patients with metastatic disease (*n* = 52) or secondary malignancies (*n* = 60), patients without surgery after nRCT (*n* = 4), as well as those receiving neo-adjuvant radiation without concurrent chemotherapy (*n* = 1) were excluded. The remaining 423 received nRCT and subsequent surgical resection and were included in further analysis (Appendix A). 

Demographic characteristics as well as clinical, pathological, and laboratory data were obtained from paper and electronic medical records. The distance to the anal verge was measured by means of either colonoscopy or rigid proctoscopy and additional digital rectal examination. The preoperative depth of tumor invasion was assessed by pelvic MRI, endorectal ultrasound and/or CT scan. Staging was performed according to the edition of the Union for International Cancer Control (UICC) classification valid at the time of rectal cancer diagnosis (6th edition for cancer diagnosis from 2004–2009 and 7th edition for 2010–2015) [26,27]. The used versions did not differ in terms of the primary tumor stage.

Treatment decisions were based on the recommendations of the multidisciplinary tumor board. Radiotherapy was performed with high-energy photons (6 or 18 MeV) up to a total dose of 45–46 Gy in 23–25 fractions of 1.8 or 2 Gy/fraction (5 days/week) prescribed to the 95% isodose. The radiation method consisted of either three-dimensional conformal radiotherapy delivered in a four-field technique or intensity-modulated radiotherapy (IMRT), including volumetric modulated arc therapy (VMAT). All patients received concurrent fluoropyrimidine-based chemotherapy that was performed as a continuous intravenous infusion with 5-fluoruracil (1000 mg/m^2^) during the first and last week of radiotherapy or as oral administration of capecitabine (1650 mg/m^2^ daily) on each day of radiation treatment. After a median interval of 7 weeks, TME or abdominoperineal rectum resection (APR) was performed. Adjuvant 5-fluorouracil based chemotherapy was administrated in 224 patients (53%).

The CRP were obtained as part of a routine clinical evaluation prior to the start of nRCT and analyzed using the standard clinical testing methodology. Additional parameters of interest assessed in the present study included age, sex, smoking status, body mass index (BMI), clinical T-stage (cT), clinical lymph node involvement, and tumor grade. 

Follow-up examinations were performed in regular intervals (3 months intervals in years 1–3, 6 months intervals in years 4–5, and 12 months intervals in years 6–10 after diagnosis) and included clinical examination, evaluation of CEA and carbohydrate antigen 19-9 (CA 19-9), radiological assessment (liver scan or ultrasound and chest X-ray every 6 months within the first three years), and colonoscopy every 2 years. 

The study complied with all the relevant national regulations, institutional policies, and in accordance with the tenets of the Declaration of Helsinki, and has been approved by the review board of the Medical University Graz (approval number: EK 28-046 ex 15/16, approval date 21 October 2015). As this is a retrospective non-intervening study, informed consent from the study participants was not required.

The primary endpoint, RFS, was calculated from the start of treatment to the date of locoregional or distant tumor recurrence. OS was defined as the time from the start of treatment to the date of death of any cause, LC was calculated from initiation of treatment to the development of local recurrence and/or regional lymph node metastases, and MFS was defined as the time from the start of treatment to the development of distant metastases. The clinical endpoints were calculated using the Kaplan–Meier method and log-rank tests were applied for statistical comparisons between Kaplan–Meier curves. Non-parametric tests (Mann–Whitney U Test, Kruskal–Wallis Test, and Spearman correlation) were applied to evaluate the association between the pre-treatment CRP level and clinico-pathological parameters. Univariate Cox proportion analysis was performed to determine the influence of an elevated CRP level and other clinico-pathological and laboratory factors such as age at diagnosis, sex, BMI, smoking status, tumor grade, tumor stage, nodal involvement, LDH, CEA, CA19-9 on OS, LC and RFS. Hazard ratios (HRs) estimated from the Cox proportion analysis were reported as relative risks with corresponding 95% confidence intervals (CIs). Multivariate Cox proportion analyses were conducted to evaluate the impact of potential confounders and included parameters with a *p*-value < 0.20 in univariate analyses. To evaluate the relationship between baseline clinico-pathological and laboratory parameters and tumor response after nRCT, logistic regression analysis was applied. Receiver operating characteristic (ROC) curve analysis was performed to estimate the optimal CRP cutoff value as reported previously [28,29]. The optimal cutoff value was determined as the point on the ROC curve that maximizes the Youden Index.

All statistical analyses were performed using the Statistical Package for Social Sciences version 26.0 (SPSS Inc., Chicago, IL, USA). A two-sided *p* < 0.05 was considered statistically significant. 

## 3. Results

### 3.1. Analysis at Baseline

Data from 423 rectal cancer patients were analyzed. Patient characteristics are presented in Table 1. The pre-treatment CRP level was significantly correlated with body mass index (BMI, *p* = 0.005) and initial tumor stage (T1/2 vs T3 vs T4, *p* < 0.001), and the involvement of the circumferential resection margin defined on the magnetic resonance imaging (mrCRM, *p* = 0.048); in addition, a significant association between the pre-treatment and post-treatment CRP level was observed (*p* < 0.001).

No significant correlations between the pre-treatment CRP level and the remaining baseline patient characteristics including patient age (*p* = 0.055), sex (*p* = 0.790), co-morbidities (*p* = 0.849), smoking status (*p* = 0.112), tumor site (*p* = 0.379), clinical stage (*p* = 0.350), nodal involvement (*p* = 0.343), tumor grade (*p* = 0.940), extramural venous involvement (EMVI, *p* = 0.844), lactate dehydrogenase (LDH, *p* = 0.620), carcinoembryonic antigen (CEA, *p* = 0.196), and carbohydrate antigen (CA 19-9, *p* = 0.175) were detected.

### 3.2. Surgical Parameters

After a median time of 7 weeks following nRCT, definitive surgical resection was performed. A total of 315 patients (74.5%) underwent TME, and 108 patients (25.5%) had APR. In 405 patients (95.7%), complete tumor resection was performed, and in 18 patients (4.3%), tumor resection was microscopically incomplete. 

The pathologic tumor stage after nRCT was ypT0 in 66 (15.6%), ypT1 in 29 (6.9%), ypT2 in 119 (28.1%), ypT3 in 189 (44.7%), and ypT4 in 20 patients (4.7%). Pathologic nodal stage was ypN0 in 293 (69.3%), ypN1 in 79 (18.7%), ypN2 in 50 (11.8%), and ypN3 in 1 of the cases (0.2%). 

Downstaging (pathological stage after nRCT < clinical stage) was detected in 231 out of 423 patients (56.4%), 53 patients (12.5%) had a complete response (ypT0 ypN0). In 184 patients (43.5%), a good response defined as ypT0-2 ypN0 was achieved. Pathological downstaging was significantly influenced by the pre-treatment clinical lymph node involvement (*p* = 0.003) and clinical stage (*p* = 0.002) whereas age (*p* = 0.335), sex (*p* = 0.840), co-morbidities (*p* = 0.472), BMI (*p* = 0.632), smoking status (*p* = 0.461), tumor site (*p* = 0.099), clinical tumor stage (*p* = 0.963), tumor grade (*p* = 0.443), mrCRM (*p* = 0.442), EMVI (*p* = 0.999), LDH (*p* = 0.533), CEA (*p* = 0.373), CA 19-9 (*p* = 0.119), as well as pre-treatment CRP (*p* = 0.634) and post-treatment CRP levels (*p* = 0.121) were not significantly associated with downstaging.

The pre-treatment tumor stage was a significant predictor of complete response (*p* = 0.007), and none of the remaining baseline parameters were significantly associated with a complete response after nRCT. Furthermore, pre-treatment tumor site (*p* = 0.007), tumor stage (*p* = 0.027), CEA (*p* = 0.005), and CA 19-9 (*p* = 0.043) were significantly associated with a good response. 

### 3.3. Outcome

The median follow-up time was 73 months (mean 77.3 ± 1.9 months). During this period, a total of 63 patients (14.9%) had died, 27 patients (6.4%) developed loco-regional recurrence, 69 patients (16.3%) had distant metastases, and 78 patients (18.4%) loco-regional recurrence and/or distant metastases. The 3- and 5- year estimates for RFS were 82.6% and 70.5%, the 3- and 5- year LC estimates were 94.5% and 90.8%, the 3- and 5- year MFS survival were 88.9% and 86.1%, and the 3- and 5- year OS probabilities were 92.0% and 80.3%, respectively.

### 3.4. Predictors of Outcome

In univariate analysis, the pre-treatment CRP level was significantly associated with RFS (HR 1.015, 95% CI 1.006–1.023; *p* < 0.001), LC (HR 1.015, 95% CI 1.004–1.027; *p* = 0.009), MFS (HR 1.014, 95% CI 1.004–1.023; *p* = 0.004), and OS (HR 1.016, 95% CI 1.007–1.024; *p* < 0.001). Furthermore, univariate analysis identified the mrCRM and CEA as a significant prognostic factor for RFS (HR 2.082, 95% CI 1.106–3.919; *p* = 0.023 and HR 1.005, 95% CI 1.002–1.008; *p* < 0.001). Univariate analysis also revealed a significant association of the mrCRM (HR 2.089, 95% CI 1.052–4.147; *p* = 0.035) and CEA (HR 1.006, 95% CI 1.003–1.008; *p* < 0.001) with MFS. Additionally, age and CEA were significantly associated with OS in univariate analysis (HR 1.039, 95% CI 1.013–1.066; *p* = 0.003 and HR 1.005, 95% CI 1.002–1.008; *p* < 0.001). None of the remaining pre-treatment patient and treatment characteristics were associated with RFS, LC, MFS, and OS in univariate analysis (Table 2). 

In multivariate analysis that included parameters with a *p*-value < 0.20 in univariate analysis, the pre-treatment CA 19-9 (HR 1.004, 95%CI 1.001–1.006; *p* = 0.010) and CRP levels (HR 1.013, 95%CI 1.001–1.025; *p* = 0.036) were identified as significant prognostic factors for RFS; furthermore, the pre-treatment CRP level remained an independent prognosticator for LC (HR 1.014, 95% CI 1.001–1.027; *p* = 0.031) and MFS (HR 1.013, 95% CI 1.000–1.027; *p* = 0.046) in multivariate analysis. In addition, multivariate analysis revealed a significant association of patient age (HR 1.040, 95% CI 1.010–1.071; *p* = 0.010), pre-treatment CEA (HR 1.004, 95% CI 1.001–1.007; *p* = 0.015), and CRP level (HR 1.015, 95% CI 1.005–1.026; *p* = 0.004) with OS.

**Figure 1:**
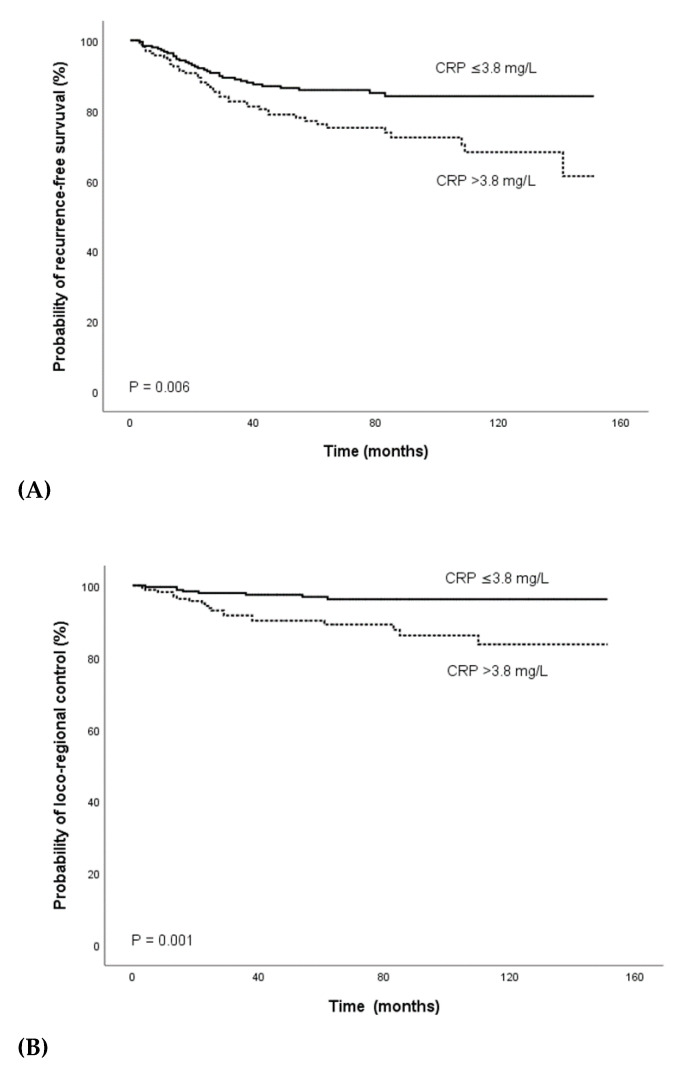

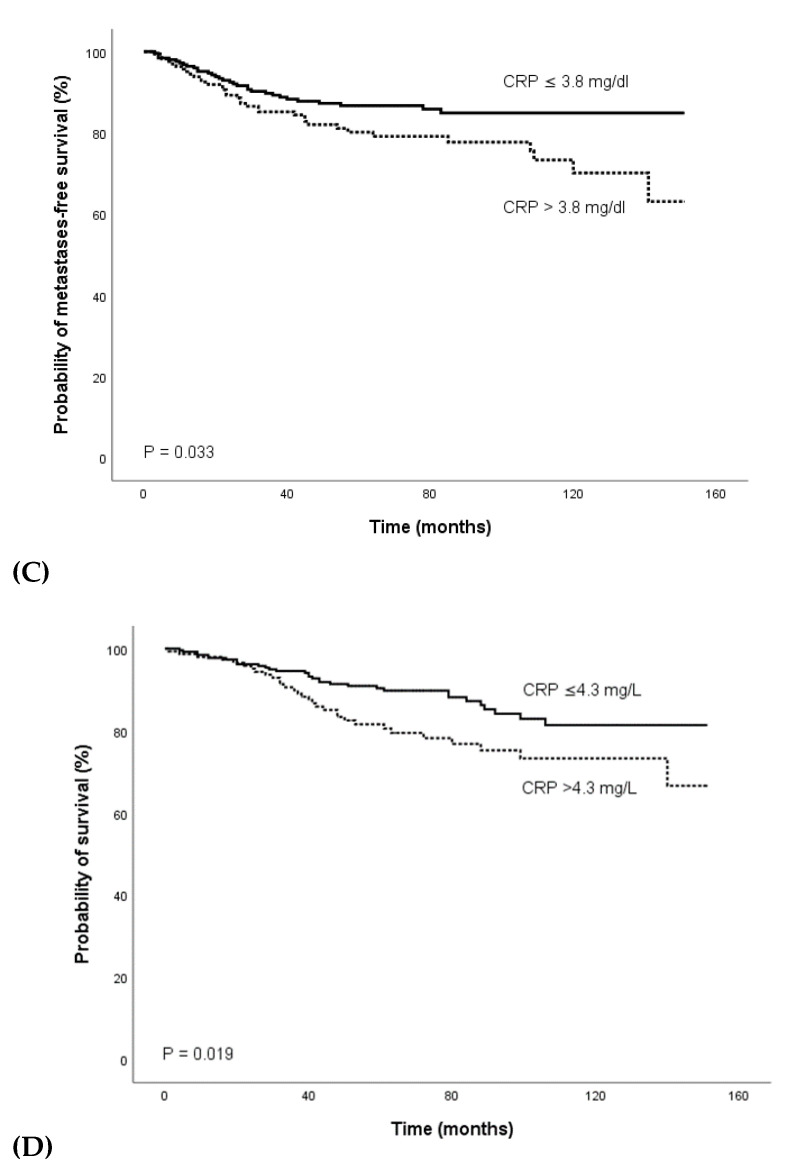
Kaplan–Meier curves for (**A**) recurrence-free survival, (**B**), loco-regional control, (**C**) metastases-free survival, and (**D**) overall survival, categorized by the pre-treatment CRP level. Abbreviation: CRP, C- reactive protein.

Using ROC curve analysis, a pre-treatment CRP cutoff value of 3.8 mg/L was determined to be optimal for both PFS and LC. Overall, there were 254 patients (60%) with a pre-treament plasma CRP level ≤3.8 mg/L and 169 patients (40%) with a CRP level >3.8 mg/L. In the Kaplan–Meier analysis, decreased PFS (*p* = 0.006) and LC (*p* = 0.001) was detected for patients with a pre-treatment CRP level >3.8 mg/L (Figure 1A,B).

For OS, ROC curve analysis showed that a CRP level of 4.3 mg/L was optimal to discriminate patient survival and death. There were 272 patients (64.3%) with a CRP level ≤ 4.3 mg/L and 151 patients (35.7%) with a CRP level > 4.3 mg/L. Kaplan–Meier analysis demonstrated a significantly decreased OS for patients with a pre-treatment CRP level > 4.3 mg/L (Figure 1C).

## 4. Discussion

The establishment of easily determinable and objective predictive factors for the outcome is critical for improving treatment strategies. In the present study, we examined the prognostic impact of the pre-treatment plasma CRP level in patients with locally advanced rectal cancer treated with nRCT and detected a significant relationship between an elevated pre-treatment CRP level and a decreased RFS, LC, MFS, and OS in univariate and multivariate analysis. These data suggest that a high pre-treatment CRP level may identify patients with poor loco-regional control, a high risk for developing distant metastases, and a poor overall survival outcome. 

There is strong evidence for the role of inflammation in cancer development, progression, and metastasization [12,30]. Most solid malignant tumors trigger an intrinsic inflammatory response that builds up a protumorigenic microenvironment [14]. Various oncogenes have been shown to induce a transcriptional program that leads to remodeling of the tumor microenvironment through the recruitment of leukocytes and expression of tumor-promoting chemokines and cytokines [31,32]. Furthermore, solid malignancies become oxygen- and nutrient-deprived, resulting in necrotic cell death and in an inflammatory response promoting angiogenesis and the synthesis of additional cytokines [33]. Cytokines such as interleukin (IL)-6, tumor necrosis factor (TNF)-a, transforming growth factor (TGF)-ß, and epithelial growth factor receptor (EGFR) ligands control the immune and inflammatory milieu and also have direct effects on cancer cell growth and survival through the activation of various downstream effectors [14,33].

Cancer- related pro-inflammatory cytokines, in particular IL-1 and IL-6, have been linked with the stimulation of CRP production; thus, increased CRP levels may represent an inflammatory microenvironment that supports tumor angiogenesis, proliferation, growth, and metastasization [34,35,36,37,38].

There is rising evidence supporting the prognostic role of the plasma CRP level in different cancer entities, including localized and metastatic CRC [39,40,41,42,43,44,45,46]. In addition, pre-therapeutic indices of systemic inflammation based on CRP and albumin have been found to provide prognostic information in CRC patients. However, only a few studies have investigated the prognostic significance of the plasma CRP level in rectal cancer. Kim et al. analyzed the prognostic role of the pre-operative CRP level determined in 125 patients after completion of nRCT and detected a significant association of an elevated pre-operative CRP level with DFS and CSS [47]. In contrast, Giessen et al., who also evaluated the prognostic role of the pre-operative CRP level in rectal cancer patients, did not detect a significant relationship between the CRP level and outcome [48].

In patients with locally advanced rectal cancer treated with nRCT, data on the prognostic significance of the pre-treatment plasma CRP level are very limited. Buijsen et al. investigated various pre-treatment biomarkers, including the plasma CRP level, as predictive factors for tumor response after nRCT, but did not detect a significant relationship between the pre-treatment CRP level and complete or good response after nRCT [25]. However, data on the relationship between the pre-treatment CRP level and the survival outcome are not provided. To our knowledge, to date, only 1 series has examined the prognostic role of the pre-treatment plasma CRP for survival outcomes in rectal cancer patients treated with nRCT. Toiyama et al. analyzed the prognostic impact of the pre-treatment CRP level in a cohort of 84 patients treated with nRCT and subsequent TME and identified an elevated pre-treatment CRP level as a significant prognostic factor for poor OS and DFS [24].

In the present study, univariate analysis identified a CRM > 1mm as a significant prognostic factor for improved RFS and MFS, but did not reach significance in multivariate analysis. Our data are in line with the results of Nikberg et al. who also detected that the CRM is not an independent factor for RFS [49]. Furthermore, pre-treatment clinical parameters such as tumor stage or tumor site were associated with tumor response; however, a significant relationship with survival outcomes was not detected. Similar findings have been obtained by Toiyama et al. who also were unable to detect a significant association between pre-treatment clinical stage and disease-free survival or OS [24]. In addition, we did not detect a significant relationship between the pre-treatment nodal stage and outcome. This finding might probably be explained by the limited accuracy achieved with current imaging techniques. For instance, Brouwer et al. reported nodal understaging in 24% of rectal cancer patients staged as cN- and overstaging in 44% of patients staged as cN+ that may result in patients’ under- or overtreatment [50]. Thus, the identification of additional prognostic factors determined at the baseline is particularly important for improvements in the treatment decision-making process. 

In our study, the pre-treatment plasma CRP level was identified as a significant prognostic factor in rectal cancer patients treated with nRCT. There is evidence that CRP itself exerts pro-inflammatory and tumor-promoting effects [51]. It has been shown that CRP may induce the expression of various molecules involved in cell-cell and cell-matrix adhesion as well as the expression of matrix metalloproteinases (MMPs) that are essential for the degradation of the basement membrane and extracellular matrix, a key step for tumor growth and metastasization [52,53]. Additionally, CRP may promote tumor growth by protecting tumor cells from apoptosis [54]. 

CRP is a significant marker of inflammation that is known to represent a critical component of tumor development and progression. Several agents affecting the CRP level have been described. Cyclooxygenase inhibitors, platelet aggregation inhibitors, lipid-lowering agents, antioxidants, and angiotensin-converting enzyme inhibitors may reduce serum levels of CRP [55]. Anti-inflammatory treatment leading to the reduction of the CRP level has been proposed to represent an effective therapeutic strategy for cancer. Previous studies have shown a significantly lower incidence of colon cancer in individuals taking aspirin and non-steroidal anti-inflammatory drugs (NSAIDs) [56,57]. Because inflammatory factors contribute to the initiation and progression of various types of cancer, NSAIDs are suggested to not only reduce the incidence but also prevent the progression of cancer by suppressing various inflammatory pathways [58]. Furthermore, NSAIDs may inhibit cancer progression via the induction of tumor cell apoptosis, protection, and repair of DNA damage, and suppression of platelet activity [59]. Statin use has also been shown in several preclinical and clinical studies to demonstrate anticancer properties in several preclinical and clinical studies [60]. Furthermore, in rectal cancer, statin use was predictive of a pathologically complete response in nRCT [61].

Worse nutritional status is associated with increased morbidity, mortality, and prolonged hospitalization and may copy cancer-related processes. In unresectable or relapsed colorectal cancer, a strong correlation between malnutrition and overall survival has been reported [62]. Furthermore, the loss of muscle mass during chemotherapy has been associated with lower rates of survival in patients with metastatic CRC [63]. In our cohort, no association between BMI and survival could be observed.

To the best of our knowledge, the present study currently represents the largest one analyzing the prognostic role of the pre-treatment CRP level in patients with locally advanced rectal cancer treated with nRCT. Major strengths include the well-defined, homogenously treated study cohort and the relatively long follow-up time. 

Nevertheless, some limitations of the present study have to be taken into account. Due to the retrospective study design, a selection bias cannot be fully excluded. Furthermore, CRP is a non- specific marker of inflammation and might be influenced by several conditions such as bacterial or viral infection, inflammatory diseases, connective tissue disorders, severe stress, and medical treatments. In view of the lack of a standardized cut-off value, ROC curve analysis was applied to determine optimal cut-off levels. For OS, the cut-off value determined using ROC analysis was different when compared to the cut-off value for the remaining endpoints. This finding might be explained by the fact that OS is a non-cancer-specific endpoint that is influenced by several other conditions or diseases. However, future investigations are necessary for the validation of the cut-off levels presented in our study.

Beside CRP, other inflammation-related parameters, such as white blood cell count, neutrophil/lymphocyte ratio, or erythrocyte sedimentation rate, are potential biomarkers for outcome in rectal cancer patients [64,65]. Further studies are required to establish whether one distinct inflammatory marker or a combination of several markers yields the highest predictive power in this patient group. 

In view of the limitations of this study, our results have to be interpreted cautiously until validated in additional prospective large-scale studies before firm conclusions about the role of the pre-treatment CRP level for prognosis in patients with locally advanced rectal cancer can be drawn. Nonetheless, even considering these limitations, our data indicate that a high plasma CRP level is an independent prognostic factor for RFS, LC, MFS, and OS in locally advanced rectal cancer patients. If confirmed by additional studies, determination of the pre-treatment CRP level could provide additional prognostic information and contribute to the identification of patients who might be candidates for a more aggressive local or systemic treatment approach or the administration of anti-inflammatory drugs. 

## 5. Conclusions

An increased pre-treatment plasma CRP level seems to represent an independent prognostic factor for RFS, LC, MFS, and OS in rectal cancer patients treated with nRCT. If the present findings are replicated in future studies, determination of the pre-treatment plasma CRP level may help to obtain a more precise individual risk profile and contribute to the tailored treatment of rectal cancer patients. 

## Figures and Tables

**Table 1:** Summary of baseline patient characteristics.

Criterion	Value
Number of patients	423
Sex	
Male Female	279 (66.0%) 144 (34.0%)
Age; median (mean ± SD)	66.00 (64.7 ± 11.0)
BMI; median (mean ± SD)	26.1 (26.4 ± 4.40)
Co-morbidities	
Yes	91 (21.5%)
No	328 (77.5%)
Smoking status	
Former * or never Current	357 (84.4%) 65 (15.4%)
Tumor site	
< 6 cm ab ano 6–18 cm ab ano	235 (55.6%) 188 (44.4%)
Tumor grade	
G1/2 G3/4	396 (93.6%) 27 (6.4%)
Clinical tumor stage	
T1/2 ** T3 T4	20 (4.7%) 358 (84.6%) 45 (10.6%)
Clinical nodal involvement	
Yes No	246 (58.2%) 177 (41.8%)
Clinical stage	
Stage II Stage III	178 (42.1%) 245 (57.9%)
MRI-defined involvement of CRM ***	
Yes	49 (11.6%)
No	190 (44.9%)
Extramural venous involvement ***	
Yes	2 (0.5%)
No	235 (55.6%)
LDH, median (mean ± SD)	174.0 (186.9 ± 56.5)
CEA, median (mean ± SD)	3.3 (9.6 ± 44.6)
CA 19-9, median (mean ± SD)	8.7 (26.7 ± 70.7)
CRP pre-treatment, median (mean ± SD)	2.8 (7.7 ± 16.8)
CRP post-treatment, median (mean ± SD	4.2 (7.5 ± 11.0)

* Former smoking was defined as tobacco abuse before or until the start of treatment. ** Because there was only one T1 tumor, T1 and T2 were grouped together. *** Radiographic parameters were available in 57% of patients. Abbreviations: BMI, body mass index; LDH, lactate dehydrogenase; CEA, carcinoembryonic antigen; CA 19-9, carbohydrate antigen; CRP, C-reactive protein; MRI, magnetic resonance imaging; CRM, circumferential resection margin; SD, standard deviation.

**Table 2:** Univariate analysis of baseline parameters for the prediction of recurrence-free survival, loco-regional control, metastases-free survival, and overall survival.

	Recurrence-Free Survival		Loco-Regional Control		Metastases-Free Survival		Overall Survival		
**Criterion**	HR (95% CI)	*p*-value	HR (95% CI)	*p*-value	HR (95% CI)	*p*-value		HR (95% CI)	*p*-value
Sex									
Female Male	1 0.906 (0.567–1.447)	0.679	1 1.554 (0.657–3.675)	0.316	1 0.840 (0.514–1.373)	0.487		1 1.458 (0.836–2.543)	0.184
Age (continuous)	1.001 (0.980–1.022)	0.914	0.995 (0.961–1.029)	0.760	1.001 (0.979–1.023)	0.934		1.039 (1.013–1.066)	0.003
BMI (continuous)	0.963 (0.903–1.027)	0.249	0.954 (0.856–1.064)	0.401	0.977 (0.914–1.044)	0.488		1.000 (0.937–1.067)	0.998
Co-morbidities									
No Yes	0.982 (0.556–1.733)	0.949	0.878 (0.332–2.322)	0.794	1.155 (0.648–2.058)	0.625		1.475 (0.845–2.575)	0.172
Smoking status									
Former/never Current	1 1.492 (0.847–2.629)	0.166	1 1.724 (0.696–4.275)	0.239	1 1.583 (0.878–2.855)	0.127		1 0.751 (0.342–1.648)	0.475
Tumor site									
< 6cm ab ano 6–18 cm ab ano	1 0.981 (0.622–1.548)	0.935	1 0.733 (0.335–1.600)	0.435	1 1.143 (0.707–1.846)	0.585		1 1.439 (0.876–2.362)	0.150
Tumor grade									
G1/2 G3/4	1 0.793 (0.290–2.171)	0.652	1 0.544 (0.074–4.012)	0.551	1 0.669 (0.210–2.129)	0.496		1 0.486 (0.119–1.989)	0.316
Tumor stage									
T1/2 T3 T4	1 1.577 (0.385–6.462) 4.073 (0.930–7.828)	0.527 0.062	1 n.a. * n.a. *	0.943 0.939	1 1.441 (0.351–5.917) 3.209 (0.717–14.36)	0.612 0.127		1 0.852 (0.266–2.733) 1.450 (0.392–5.362)	0.788 0.578
Nodal involvement									
No Yes	1 1.226 (0.770–1.952)	0.390	1 1.837 (0.804–4.198)	0.149	1 1.136 (0.697–1.851)	0.610		1 0.997 (0.606–1.639)	0.990
Clinical stage									
II III	1 1.174 (0.739–1.863)	0.497	1 1.563 (0.702–3.480)	0.275	1 1.080 (0.665–1.756)	0.756		1 1.012 (0.616–1.665)	0.961
mrCRM									
No Yes	1 2.082 (1.106–3.919)	0.023	1 1.305 (0.428–3.978)	0.639	1 2.089 (1.052–4.147)	0.035		1 1.164 (0.507–2.669)	0.721
EVMI									
No Yes	1 n.a. *	0.646	1 n.a. *	0.777	1 n.a. *	0.674		1 n.a. *	0.664
LDH (continuous)	1.002 (0.998–1.006)	0.305	0.994 (0.985–1.003)	0.219	1.003 (0.999–1.007)	0.108		1.001 (0.996–1.005)	0.758
CEA (continuous)	1.005 (1.002–1.008)	<0.001	1.001 (0.993–1.010)	0.767	1.006 (1.003–1.008)	<0.001		1.005 (1.002–1.008)	<0.001
CA 19-9 (continuous)	1.002 (0.999–1.004)	0.138	1.002 (0.997–1.006)	0.527	1.002 (0.999–1.005)	0.127		1.000 (0.996–1.004)	0.918
Pre-nRCT CRP (continuous)	1.015 (1.006–1.023)	<0.001	1.015 (1.004–1.027)	0.009	1.014 (1.004–1.023)	0.004		1.016 (1.007–1.024)	<0.001
Post-nRCT CRP (continuous)	1.011 (0.993–1.030)	0.227	1.020 (0.996–1.045)	0.103	1.013 (0.995–1.032)	0.160		1.001 (0.978–1.025)	0.915

* Because of the low number of events, the calculation of HR was not applicable. Abbreviations: CI, confidence interval; HR, hazard ratio; BMI, body mass index; CEA, carcinoembryonic antigen; LDH, lactate dehydrogenase; CA 19-9, carbohydrate antigen 19-9; CRP, C-reactive protein; mrCRM, MRI-defined involvement of the circumferential margin; EVMI, extramural venous involvement; nRCT, neo-adjuvant radiochemotherapy; n.a., not applicable.

## Appendix A

**Table A1:** Inclusion and exclusion criteria for patients who were referred for neoadjuvant radiochemotherapy and retrospective analysis at our department.

Inclusion Criteria	Exclusion Criteria
Neoadjuvant radiochemotherapy with subsequent surgical resection	Metastatic disease (*n* = 52)
	Secondary malignancies (*n* = 60)
	No surgical resection (*n* = 4)
	No concurrent chemotherapy (*n* = 1)

Between 2004–2015 records of 540 patients with histological confirmed rectal cancer were identified. 423 patients received neoadjuvant radiochemotherapy and subsequent surgical resection and were included in further analysis of this study.
